# The Comparative Associations of Ultrasound and Computed Tomography Estimates of Muscle Quality with Physical Performance and Metabolic Parameters in Older Men

**DOI:** 10.3390/jcm7100340

**Published:** 2018-10-10

**Authors:** Michael O. Harris-Love, Nilo A. Avila, Bernadette Adams, June Zhou, Bryant Seamon, Catheeja Ismail, Syed H. Zaidi, Courtney A. Kassner, Frank Liu, Marc R. Blackman

**Affiliations:** 1 Muscle Morphology, Mechanics, and Performance Laboratory, Geriatrics Service, Veterans Affairs Medical Center, Washington, DC 20422, USA; blpadams@gmail.com (B.A.); seamon@musc.edu (B.S.); 34Cath@gmail.com (C.I.); 2 Milken Institute School of Public Health, The George Washington University, Washington, DC 20052, USA; 3 Research Service, Veterans Affairs Medical Center, Washington, DC 20422, USA; June.Zhou@va.gov (J.Z.); Marc.Blackman@va.gov (M.R.B.); 4 Radiology Service, Veterans Affairs Medical Center, Washington, DC 20422, USA; nilo.avila@nih.gov (N.A.A.); Syed.Zaidi2@va.gov (S.H.Z.); Courtney.Kassner@va.gov (C.A.K.); 5 Cardiovascular and Pulmonary Branch, National Heart, Lung, and Blood Institute, The National Institutes of Health, Bethesda, MD 20892, USA; 6 Department of Biochemistry & Molecular Medicine, The George Washington University, Washington, DC 20037, USA; 7 Department of Medicine, The George Washington University, Washington, DC 20052, USA; 8 Nuclear Medicine Section, Veterans Affairs Medical Center, Washington, DC 20422, USA; Frank.Liu2@va.gov; 9 Departments of Medicine and Rehabilitation Medicine, Georgetown University School of Medicine, Washington, DC 20007, USA

**Keywords:** aging, muscle tissue composition, echogenicity, sarcopenia, muscle quality, quantitative ultrasound, computed tomography, muscle strength, metabolic status, myosteatosis

## Abstract

Estimates of muscle tissue composition may have greater prognostic value than lean body mass levels regarding health-related outcomes. Ultrasound provides a relatively low cost, safe, and accessible mode of imaging to assess muscle morphology. The purpose of this study was to determine the construct validity of muscle echogenicity as a surrogate measure of muscle quality in a sample of older, predominantly African American (AA) participants. We examined the association of rectus femoris echogenicity with mid-thigh computed tomography (CT) scan estimates of intra- and intermuscular adipose tissue (IMAT), basic metabolic parameters via blood sample analysis, muscle strength, and mobility status. This observational study was conducted at a federal medical center and included 30 community-dwelling men (age, 62.5 ± 9.2; AA, *n* = 24; Caucasian, *n* = 6). IMAT estimates were significantly associated with echogenicity (*r* = 0.73, *p* < 0.001). Echogenicity and IMAT exhibited similar associations with the two-hour postprandial glucose values and high-density lipoproteins values (*p* < 0.04), as well as grip and isokinetic (180°/s) knee extension strength adjusted for body size (*p* < 0.03). The significant relationship between ultrasound and CT muscle composition estimates, and their comparative association with key health-related outcomes, suggests that echogenicity should be further considered as a surrogate measure of muscle quality.

## 1. Introduction

The maintenance of adequate lean body mass (LBM) with increasing age remains an important determinant of physical health, especially in older individuals who undergo surgical procedures or have extended hospital stays [1,2]. It has been known for nearly two decades that poor skeletal muscle tissue composition is often a better predictor of muscle performance than LBM in ambulatory older adults [3,4]. Nevertheless, this research insight has not been effectively integrated into medical practice. This is largely due to the cost and access constraints associated with the use of computed tomography (CT) and magnetic resonance imaging (MRI) for the assessment of body composition or age-related muscle dysfunction [5,6]. Given the importance of both LBM and skeletal muscle tissue composition to understanding and assessing sarcopenia, many investigators have proposed the use of diagnostic musculoskeletal ultrasound as an alternative imaging approach [7,8,9].

Muscle echogenicity has been previously proposed as a method to estimate muscle tissue composition [10,11]. Quantitative ultrasound measures of echogenicity denote image brightness caused by the reflection of soundwaves and influenced by sound beam characteristics and tissue density [12]. While echogenicity measures have been associated with intra- and intermuscular fat in older adults and children with neuromuscular disease, hyperechoic tissue may also mark other non-contractile elements of skeletal muscle [13,14,15]. However, findings utilizing biopsied muscle tissue reveal that echogenicity has a significant positive association with the proportion of intramuscular fat in a sample of people with neuromuscular disease [16]. Recent data also suggest that echogenicity is associated with extramyocellular lipids based on mid-thigh T1-weighted MRI and ^1^H magnetic resonance spectroscopy (^1^H MRS) data obtained from young and old adults [17]. Additionally, preliminary reports indicate that echogenicity estimates of anterior mid-thigh muscles may be associated with corresponding CT scan attenuation at the region of interest (ROI) in a sample of community-dwelling women [13].

The use of quantitative ultrasound to measure muscle echogenicity has been employed to aid the diagnosis of neuromuscular disease [14], estimate post-exercise intervention changes in muscle composition [18], and identify adults with age-related muscle dysfunction [19]. Importantly, there is strong evidence for the inverse association between muscle echogenicity and muscle strength in both older men and women [20,21], and early findings suggest that rectus femoris echogenicity may aid the clinical identification of sarcopenia [19]. Relative measures of strength are increasingly recognized as a domain of “muscle quality” [11,22,23]. Consequently, surrogate measures of muscle quality such as echogenicity may help to identify those with an elevated risk of strength impairment and functional decline. Estimates of muscle strength and tissue composition have demonstrated greater prognostic value than LBM levels regarding health-related outcomes [23]. In addition, extramyocellular (i.e., intra- and intermuscular adipose tissue) and intramyocellular lipids may partially contribute to declines in muscle strength that are not attributable to a loss of muscle mass alone [3,17]. Therefore, practical and reliable methods of detecting poor muscle tissue composition that affect muscle performance, but precede the appreciable decline of LBM, have clinical value. Given the increased risk for hospitalization and cardiovascular disease mortality associated with poor muscle tissue composition [23,24], quantitative ultrasound morphology measures may have a role in the management of patients with certain geriatric syndromes [9].

The purpose of this study was to determine the construct validity of muscle echogenicity as a surrogate measure of muscle quality in a sample of older, predominantly African American men. Construct validity was examined based on the association of rectus femoris echogenicity with mid-thigh CT scan estimates of intra- and intermuscular adipose tissue (IMAT), basic metabolic parameters via blood sample analysis, muscle strength, and mobility status.

## 2. Methods

### 2.1. Study Design

This study featured a prospective, single group, observational design to evaluate the construct validity of estimating muscle tissue composition using quantitative ultrasound. The study included a sample of convenience and was conducted in a clinical environment at a federal medical center. Data were collected by staff in the Physical Medicine and Rehabilitation, Pathology and Laboratory Medicine, Research Service Laboratory and Nuclear Medicine and Radiology service groups at the Veterans Affairs Medical Center in Washington, DC (DC VAMC).

### 2.2. Study Participants

Community-dwelling veteran men were screened at the medical center for potential enrollment into the Age-Related Muscle Dysfunction Screening Study (ARMS Study I). The ARMS Study I was approved separately by the DC VAMC Institutional Review Board and the Research and Development Committee. Inclusion criteria for study enrollment included being an ambulatory male veteran between 45 and 85 years of age. Exclusion criteria included recent hospitalization (within 3 months), uncontrolled cardiovascular disease, limb amputation, medical conditions that result in edema, and musculoskeletal or neurological disorders (e.g., cerebrovascular accident resulting in hemiparesis or intrinsic muscle disease) that are associated with muscle atrophy.

A clinical research nurse screened 104 study candidates at the medical center and 30 individuals qualified for participation in the study. Screening failures during study recruitment were due to uncontrolled cardiovascular or metabolic disease (*n =* 38), recent hospitalizations (*n =* 16), conditions that may confound dual-energy X-ray absorptiometry (DXA) findings (e.g., edema, major joint replacement, or body stature; *n =* 7), age outside of the study inclusion range (*n =* 5), and qualified individuals unable or unwilling to fully participate for other reasons (*n =* 8). Upon obtaining written informed consent, all participants completed the imaging examinations, strength tests, functional assessments, anthropometric measures, and blood sampling. Outcome measures were completed over the course of 2 visits (with a span of 10 days or less between visits), and imaging procedures were always performed on a separate visit from the physical performance tests.

### 2.3. Outcome Measures

The clinical reference standards for body composition and muscle tissue composition estimates in this study were DXA and CT scanning, respectively [5,25,26]. The CT scans, and corresponding ultrasound scans, were taken at the anterior mid-thigh given the previous use of this site to estimate IMAT [3,27,28,29,30]. The physical status of the participants was represented by performance-based assessments. The primary measures of general physical performance and mobility were peak force from hand grip dynamometry and gait speed derived from the 10-m walk test given the extensive use of these assessments in clinical trials involving ambulatory older adults [26,31]. In addition, peak knee extensor torque was used to characterize muscle performance of the anterior thigh muscle groups included in the imaging examinations [29,30,32]. Basic biomarkers of metabolic health were assessed via a lipid panel and fasting insulin/glucose levels taken from a blood sample [33]. 

#### 2.3.1. Tissue and Body Composition Analyses

A portable, diagnostic ultrasound device (SonoSite M-Turbo 1.1.2; SonoSite, Inc., Bothell, WA, USA) with a 13.6 MHz linear array transducer was used for B-mode scanning and image acquisition. Time gain compensation, and well as near field/far field gain, remained at the manufacturer default settings for musculoskeletal scanning. The field of view was adjusted for each participant to optimally capture the ROI for the rectus femoris. Water-soluble transmission gel was used during scanning to maintain adequate acoustic contact with the skin surface, and minimal examiner pressure was used during the scanning to avoid tissue deformation prior to image capture. The dominant-side scanning site and procedures used in this study have been previously reported by the authors [19]. Briefly, surface anatomy landmarks were identified at the anterior superior iliac spine and the superior pole of the patella. The bisected distance (cm) between the surface landmarks were measured using a non-elastic weighted tape measure and marked with an indelible ink pen. Quantitative ultrasound imaging was performed with the participants seated with their feet on the floor and their elbows, hips, and knees were positioned at 90° of flexion. All scanned images were obtained and measured 3 times within the fascial boarders of the muscle. The ROI selection and echogenicity measures of the rectus femoris via grayscale histogram analysis were completed using ImageJ (version 1.48; National Institutes of Health, Bethesda, MD, USA) [34]. A single clinical investigator with over 10 years of quantitative ultrasound experience acquired the scanned images and obtained the echogenicity measures (echogenicity expressed as grayscale levels (GSL) within a unitless range of 0–255). This scanning procedure and image analysis approach has been shown to have good reliability in our facility (lower bound 95% confidence interval (CI) intraclass correlation coefficient (ICC_2,*k*_) = 0.97–0.99, *p* < 0.001, standard error of measurement, SEM = 1.05 grayscale levels) [27].

Estimates of IMAT were obtained via CT imaging at the mid-thigh (Siemens 64; SD = 0.2 Hounsfield units (HU), coefficient of variation, CV = 0.005). A single 10 mm axial image slice (120 kVp, 200 to 250 mA) of the thigh was obtained on the dominant limb. The scanning site for ultrasound and CT imaging were at a common location identified during the ultrasound examination. The mid-thigh target location marked with indelible ink was covered with a radiopaque marker prior to the CT scanning session. An anterior–posterior scout scan of the entire femur was completed followed by an axial scan featuring the marker to verify the ROI. Upon verification of the scanning location, a second image was acquired without the marker for the subsequent image analyses. A radiologist and study co-investigator (N.A.) performed the initial review of the CT scans. Image processing and analysis of the CT scans for tissue segmentation was performed using ImageJ. The primary investigator (M.H.L) completed the tissue segmentation procedure in consultation with the radiologist. The deep fascial layer was digitized prior to calculating estimates for the proportion of cross-sectional area (CSA) subcutaneous fat, and IMAT [3]. All tissue composition estimates were adjusted to not include pixel values derived from cortical bone, medullary fat, or potential confounders for LBM within the dermal layers. Tissue analyzed in the ROI within +31 to +100 HU range was classified as normal density muscle, and tissue in the 0 to +30 HU range was classified as low-density muscle affected by intramuscular adipose tissue [35]. Tissue in the −190 to −30 HU range was denoted as adipose tissue with further classification as subcutaneous fat or intermuscular fat based on its location relative to the deep fascial layer and intermuscular septa of the thigh musculature [3].

Estimates of LBM via whole body DXA imaging using a GE Lunar iDXA machine (GE Medical Systems Ultrasound and Primary Care Diagnostics, LLC, Madison, WI, USA) were completed by a trained radiology technician. Height (cm) was measured with a stadiometer and body mass (kg) was measured with a balance scale prior to body composition testing. Body mass index (BMI) was calculated as weight (kg)/height (m^2^). Total body fat values were recorded, and LBM estimates were calculated as the sum of fat-free mass in the arms and legs (aLM) and scaled to height (aLM/ht^2^). The preparation and positioning of the participant was performed according to the DC VAMC Radiology Service testing procedures. All DXA scans were obtained on the same day as the quantitative ultrasound session.

#### 2.3.2. Blood Sampling: Metabolic Parameters

Following an overnight fast, the metabolic parameters were measured from venous blood samples withdrawn from an antecubital vein. Serum glucose and lipid concentrations were measured by routine methods in the Clinical Laboratory Improvement Amendments certified Clinical Pathology Laboratory of the Washington DC VA Medical Center. Serum insulin was measured in the Research Laboratory of Geriatric Endocrinology and Metabolism.

##### Oral Glucose Tolerance Test (OGTT)

Venous blood samples (10 mL) were obtained before and 2 h after oral ingestion of a 75 g oral glucose solution. Whole blood samples were allowed to clot for 5–10 min, centrifuged for 10 min at 2000× *g*, and the resultant serum stored at −80 °C in aliquots until assayed. Interpretation of the oral glucose tolerance test (OGTT) two-hour postprandial glucose values were as follows: normal < 140 mg/dL; impaired glucose tolerance 140–199 mg/dL; diabetic ≥200 mg/dL.

##### Glucose and Insulin

Serum glucose was assayed using the hexokinase method utilizing the glucose-6-phosphate dehydrogenase and colorimetric reading. Test results were interpreted per the American Diabetes Association Diagnostic criteria: normal < 100 mg/dL, pre-diabetic 100–125 mg/dL; diabetic > 125 mg/dL. Serum concentrations of insulin were measured by enzyme-linked immunosorbent assay (ELISA) (Millipore Sigma Corporation, Catalog No. EZHI-14K) following procedures recommended by the manufacturer. This sandwich assay is based, sequentially, on: (1) capture of human insulin molecules from samples to the wells of a microtiter plates coated by a pre-titered amount of monoclonal mouse anti-human insulin antibodies and the binding of a second biotinylated monoclonal mouse anti-human antibody to the captured insulin, (2) washing away of unbound materials from samples, (3) conjugation of horseradish peroxidase to the immobilized biotinylated antibodies, (4) washing away of free enzyme conjugates, and (5) quantification of immobilized antibody-enzyme conjugates by monitoring horseradish peroxidase activities in the presence of the substrate 3,3′,5,5′-tetramethylbenzidine. The enzyme activity is measured spectrophotometrically after acidification of formed products.

All samples were assayed in duplicate, with sensitivity (1 μU/mL), range (2 μU/mL to 200 μU/mL), intra-assay CV (4.6–7.0%) and inter-assay CV (9.1–11.4%) within the limits indicated by the manufacturer. Normal fasting serum insulin concentrations are <25–30 μU/mL. Quantitative insulin sensitivity check index (QUICKI) was calculated as 1/[log(I_0_) + log(G_0_)], where I_0_ was the fasting insulin value, and G_0_ was the fasting glucose value.

##### Lipids

Lipid assays were performed on a Roche Cobas c501 System following procedures recommended by the manufacturer. Total cholesterol was measured by a timed end point method in which cholesterol esterase hydrolyzes cholesterol esters to free cholesterol and fatty acids. Free cholesterol is oxidized to cholestene-3-one and hydrogen peroxide by cholesterol oxidase. Peroxidase catalyzes the reaction of hydrogen peroxide with 4-aminoantipyrine and phenol to produce a colored quinoneimine product. The Roche cholesterol assay meets the 1992 National Institutes of Health (NIH) goal of less than or equal to 3% for both precision and bias. Normal total cholesterol level is <200 mg/dL.

The high-density lipoproteins (HDL) assay involves elective formation of water-soluble complexes between dextran sulfate and low-density lipoproteins (LDL), very low density lipoprotein and chylomicrons which are resistant to PEWG-modified enzymes used in the subsequent enzymatic steps leading to formation of a colored product. Calculated LDL levels were determined according to Friedewald’s formula. The laboratory normal HDL range was >35 mg/dL, and the range was ≤130 mg/dL for LDL.

Triglycerides are measured in a timed end-point method in which triglycerides are hydrolyzed to glycerol and free fatty acids by the action of lipase, followed by three enzymatic steps catalyzed by glycerol kinase, glycerophosphate oxidase, and horseradish peroxidase which lead to the formation of a colored product. The laboratory normal triglyceride range was 60–190 mg/dL.

#### 2.3.3. Physical Performance: Strength and Mobility

The construct of “strength” was represented by the averaged peak force or torque values scaled to body weight given the well-known influence of body size on the expression of unadjusted strength values [36,37,38]. Upper extremity strength was assessed using hand grip dynamometry (Jamar, Lafayette Instruments, Lafayette, IN, USA). The test proctor provided a demonstration of the dynamometer and allowed the participant to have one to two practice attempts prior to recording peak force data. The mean value of three trials under standardized conditions were used for data collection [39]. Grip strength is a common measure of muscle function in older adults and a preferred method of strength assessment for sarcopenia clinical trials [26]. The Jamar dynamometer has exhibited high reliability (ICCs = 0.97–0.98, *p* < 0.01) in previous studies [40]. Lower extremity strength was assessed using an isokinetic dynamometer (Biodex System 4, Biodex Medical Systems, Shirley, NY, USA). Knee extensor strength was assessed at isokinetic speeds of 60°/s and 180°/s using methods adapted from previously published protocols [41,42] with patient positioning and stabilization per the Biodex Operations Manual. Participants were oriented to the visual feedback for the torque-time curve displayed on the computer monitor, and provided with a familiarization session, prior to data collection. Strength data were compiled from the mean value of the highest three peak torque values from a five-repetition test. Previous investigators have found this form of strength assessment to be reliable (ICC = 0.92), and the standard error of the mean (SEM) is approximately 8% in older adults [42,43]. In addition, gait speed was used to characterize mobility in this study. The gait speed test is perhaps the most common screening test used for sarcopenia [26]. The 10-m walk test included in this study was recorded with a stop watch and adapted from the methods of Bohannon et al. and others [44,45].

## 3. Statistical Analysis

Descriptive statistics were used to convey the outcome measures and participant characteristics. Data were expressed as means and standard deviations if they had normal data and variance distributions. Otherwise, data were expressed as median values with the interquartile range (IQR) [46]. Inferential statistics were used to examine the construct validity of muscle echogenicity as a surrogate measure of muscle quality. Pearson product-moment correlation coefficients (PMCC, *r*) were used to determine the associations among the estimates of body composition, metabolic parameters, muscle tissue composition, and physical performance. Significant bivariate relationships between the muscle tissue composition estimates and metabolic parameters were identified using the PMCC and further examined while adjusting for the influence of known IMAT covariates: age and total body fat [47,48]. Multiple linear regression was used to assess the conditional association between estimates of muscle tissue composition and selected metabolic parameters using standardized beta coefficients. The F values from an analysis of covariance were used to assess the integrity of each multiple linear regression equation. The strength of the association among the correlation coefficients was determined using criteria noted by Portney and Watkins [46].

Independent t-tests were used to determine if preliminary cutoff values for muscle tissue composition estimates could discriminate between participants with high or low strength performance. These analyses were conducted using criterion values from the first and third tertiles of IMAT and echogenicity GSL values. This strategy was employed since there is no standard cutoff value for muscle tissue composition estimates. Additionally, our previous work suggests that the residuals associated with the bivariate relationship between GSL values and scaled strength values are largest within the second tertile [19]. It was presumed that study findings showing a significant relationship for GSL values with metabolic parameters, IMAT estimates, and physical performance would support the construct validity of muscle echogenicity as a surrogate measure of muscle quality [49]. Construct validity would be further supported by significant differences in strength values based on first and third GSL tertiles of muscle echogenicity.

Power analyses were completed using G*Power (version 3.0.10) with α set at 0.05 for all estimates. The analyses regarding the association between echogenicity and IMAT were informed by our interim cohort analysis [50] based on the *F* test model for linear multiple regressions with three predictor variables featuring a random model approach (ρ^2^ = 0.64). This yielded a β value of 0.83 and a required sample size of 15. The analyses regarding the association between echogenicity and strength were informed by preliminary data from our group [51] involving multiple scanning sites and grip strength (ρ^2^ = 0.27–0.54), which results in β values of 0.80–0.84 and a sample size range of 14–31. Study power for difference analyses were based on the *t* test model for independent groups. The observed effect sizes (*d* = 1.07–1.14) were derived from our previously reported data concerning echogenicity and grip strength in participants with and without sarcopenia [19]. These calculations yielded a sample size estimate of 28−30 participants. Statistical analyses were performed using SPSS statistical software for Windows (version 10.0, SPSS Inc., Chicago, IL, USA). Data with a non-normal distribution were log transformed prior to statistical analyses involving bivariate relationships. Levene’s test was used for analysis of differences involving data with non-normal distributions of variance, and two-tailed *p* values < 0.05 were considered significant for all inferential statistics.

## 4. Results

Our final study population consisted of 30 veterans who were primarily older African-American men (average age: 62.5 ± 9.2 years). A complete description of the participants’ characteristics is provided in Table 1. In general, our sample was overweight (BMI: 26.3 ± 3.8) and had a high total body fat percentage (BF%: 27.8 ± 7.4%). In terms of physical performance and mobility, our sample performed relatively well with both grip strength and gait speed falling within normal limits for healthy older adults [52,53]. Additionally, the average values for metabolic parameters fell within normal values for healthy individuals based on reference values used at the study site medical center.

### 4.1. Surrogate Measures of Muscle Quality and Body Composition Estimates

Muscle composition estimates served as surrogate measures of muscle quality. These estimates were derived from images obtained at the mid-thigh via CT and ultrasound scanning at the same anatomical plane of measurement. IMAT estimates from the mid-thigh CT scans were strongly associated with echogenicity measures from the rectus femoris (*r* = 0.73, *p* < 0.001). Echogenicity and IMAT both exhibited significant direct relationships with mid-thigh CSA subcutaneous fat and total body fat estimates. The relationships among surrogate measures of muscle quality and body composition estimates are reported in Table 2.

### 4.2. Surrogate Measures of Muscle Quality, Body Composition, and Metabolic Parameters

Muscle echogenicity and IMAT both exhibited significant direct relationships with the OGTT two-hour postprandial glucose values and inverse associations with high-density lipoproteins (HDL) values. Echogenicity demonstrated the strongest positive association with OGTT (*r* = 0.43, *p* = 0.018), and IMAT had the strongest negative relationship with HDL values (*r* = −0.61, *p* < 0.001). However, total body fat also exhibited significant, comparable associations with these metabolic parameters. In addition, LBM was directly associated with fasting glucose levels (*r* = −0.46, *p* < 0.023), but not with fasting insulin levels or the OGTT results (*p* > 0.05). The relationships among surrogate measures of muscle quality, body composition estimates, and general metabolic parameters are provided in Table 3.

Given the significant associations of surrogate measures of muscle quality with postprandial glucose values and HDL results, we selected these two metabolic parameters as dependent variables in multiple linear regression models to adjust for the known covariates: age and total body fat. IMAT retained a moderate association with the postprandial glucose values when accounting for age and total body fat (Age: β = −0.006, BF%: β = 0.184, IMAT: β = 0.405; F = 3.06, *p* = 0.046). IMAT demonstrated a similar association with HDL as the dependent variable in the model (Age: β = 0.237, BF%: β = −0.444, IMAT: β = −0.461; F = 7.30, *p* = 0.001). The associations for echogenicity were comparable to IMAT when examining the same metabolic parameters and covariates. Specifically, echogenicity (expressed as GSL) retained a moderate association with the HDL results when accounting for age and total body fat (Age: β = 0.166, BF%: β = −0.434, GSL: β = −0.395; F = 6.62, *p* = 0.002). We found echogenicity to be a moderate contributor to the model for postprandial glucose values with either age as a co-variate (Age: β = 0.038, GSL: β = 0.460; F = 4.10, *p* = 0.028) or total body fat as a co-variate (BF%: β = 0.162, GSL: β = 0.400; F = 4.54, *p* = 0.020). However, the model for echogenicity as predictor variable for HDL was not significant when adjusting for both age and total adiposity (F = 2.93, *p* = 0.052).

### 4.3. Surrogate Measures of Muscle Quality, Body Composition, and Physical Performance

Surrogate measures of muscle quality and LBM were generally associated with muscle strength. Echogenicity was the only outcome measure to be significantly associated with both absolute strength and strength values adjusted for body size. IMAT was only associated with adjusted strength values for hand grip and the knee extensors (180°/s), whereas LBM was only associated with absolute strength values for grip and the knee extension at both movement velocities (60°/s and 180°/s). None of the outcome measures regarding muscle tissue composition and body composition were significantly associated with customary or fast gait speed (*p* > 0.05). The relationships among surrogate measures of muscle quality, body composition estimates, and muscle strength are presented in Table 4.

### 4.4. Surrogate Measures of Muscle Quality as Discriminators of Physical Performance

We examined the ability of echogenicity and IMAT tertile values to discriminate among individuals with relatively lower and higher strength values. Significant differences in all absolute and adjusted strength values (*p* = 0.016–0.026) were observed between participants in the first and third tertiles defined by echogenicity values. Additionally, adjusted peak hand grip force (mean difference, MD: 0.10, 95% CI (0.03–0.18), *p* = 0.016) and adjusted peak knee extensor torque at 180°/s (MD: 0.11, 95% CI (0.01–0.22), *p* = 0.040) significantly differed between participants in the first and third tertiles defined by IMAT values. Tertiles determined by IMAT or echogenicity did not discriminate among individuals on gait performance (*p* > 0.05) with the exception of fast gait speeds between participants with echogenicity values in the first and third tertiles (MD: 0.45 m/s, 95% CI (0.09–0.80 m/s), *p* = 0.017). The comparison of mean muscle strength values based on the first and third tertile of echogenicity and IMAT are provided in Table 5.

## 5. Discussion

**Figure 1:**
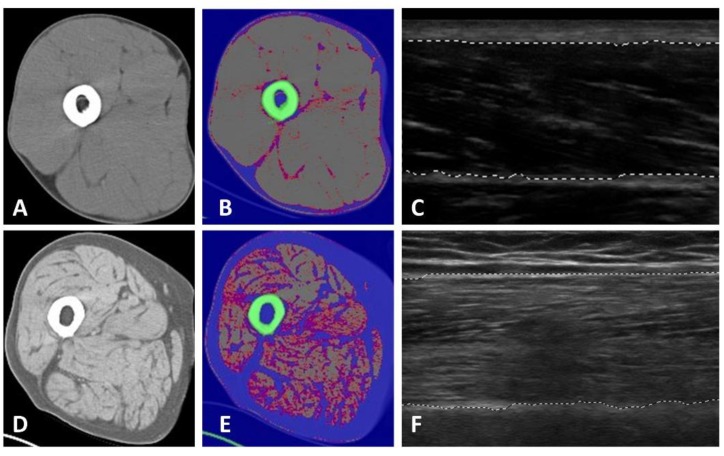
Exemplar ultrasound images of the rectus femoris and axial computed tomography CT images of the mid-thigh in two study participants. Mean grayscale measures of echogenicity at the rectus femoris of the dominant leg were derived from longitudinal ultrasound images and axial CT scans were obtained from the same anatomical plane at the mid-thigh. The images featured in Panel (**A**–**C**) depict a study participant with 15% cross-sectional fat mass and 7% intra- and intermuscular adipose tissue (IMAT). In contrast, the images in Panel (**D**–**F**) show a study participant with 66% cross-sectional fat mass and 40% IMAT (Hounsfield units: −190 to −30 for adipose tissue, 0 to +30 for low density muscle, and +31 to +100 for normal density muscle tissue).

The goal of this study was to examine the construct validity of muscle echogenicity as a surrogate measure of muscle quality in a sample of older, predominantly African American men. We reported the association between rectus femoris echogenicity GSL values and mid-thigh CSA IMAT values obtained from CT imaging (*r* = 0.73, *p* < 0.001; Figure 1). Both IMAT and echogenicity exhibited significant relationships with adjusted hand grip and knee extensor strength (180°/s), two-hour postprandial glucose values, and HDL values. The findings presented in this report support the construct validity of rectus femoris echogenicity as a surrogate measure of muscle quality and extend previously published data concerning the impact of excessive IMAT on muscle performance.

### 5.1. Surrogate Measures of Muscle Quality Are Associated with Strength and Physical Performance

Age-related changes in muscle tissue composition are associated with diminished strength and poor longitudinal health outcomes [23,30]. Foundational work concerning the link between muscle quality and physical performance was established in older adults based on CT muscle attenuation values [29,54], and later corroborated using ultrasound muscle echogenicity values [21,55]. Quadriceps muscle echogenicity has been significantly associated with peak isometric knee extensor strength even when adjusting for age, muscle size, or subcutaneous fat [20,21]. Moreover, muscle echogenicity measures of the anterior thigh musculature have been reported to be inversely related to strength and gait speed, and may discriminate between older women who are able or unable to ambulate independently [56]. Our reported findings regarding the moderate independent association of rectus femoris echogenicity with peak knee extensor torque is consistent with the findings of Fukumoto et al. [20] and Watanabe et al. [21]. Furthermore, we observed that echogenicity of the rectus femoris was consistently associated with all upper and lower extremity strength outcomes with and without adjustments for body size. In contrast, LBM was moderately related to only measures of strength unadjusted for body size. The significant association between rectus femoris echogenicity and grip strength in the current sample of older men supports our earlier reported observation in a sample of younger and older community-dwelling women [19].

Regarding functional performance, Visser and associates [29] have reported that a model of knee extensor muscle strength and mid-thigh IMAT, but not muscle cross-sectional area, predicted self-reported mobility limitations in older adults. High levels of IMAT (i.e., myosteatosis) have also been significantly associated with impaired lower extremity function in large cohorts of community dwelling adults and may help explain why decreases in LBM are not linearly related to declines in physical performance [21,54]. Furthermore, quadriceps echogenicity has been shown to be among the strongest independent variables in predictive models of timed sit-to-stand performance in a sample of sedentary older men [57]. In the current study, we observed significant differences in mobility in study participants during the fast gait testing condition when considering those in the first and third tertiles of echogenicity values, but not IMAT values. While muscle tissue composition estimates appear to be independently associated with muscle performance, it is likely that the relationship of muscle morphology with functional performance varies based on sample characteristics, selection of the functional test, and the method of tissue analysis employed [58,59,60].

### 5.2. The Comparative Use of Ultrasound, Computed Tomography (CT), and Magnetic Resonance Imaging (MRI) for Muscle Tissue Composition Estimates

The findings of this report build upon previous validation studies regarding the relationship of diagnostic ultrasound muscle morphology measures with CT estimates of tissue composition [61]. This early work was centered on the comparative use of: backscattered radio frequency signals and CT muscle density estimates; compound ultrasound image acquisition supplemented by multiple photographs to obtain muscle morphology data; use of a four-point subjective scale to assess intramuscular echogenicity [13,62,63]. Sipilä and Suominen’s [13,63] work involving older women athletes (73.7 ± 5.6 years old) from Finland showed that anterior mid-thigh echogenicity scores were inversely associated with the mean quadriceps HU values (*r* = −0.351, *p* < 0.05). We observed a stronger relationship between muscle echogenicity and CT estimates of muscle composition in our study sample than did Sipilä and Suominen, possibly because of differences in lower extremity IMAT based on sample characteristics such as sex, age, and racial/ethnic group [48,64,65]. Although the older, predominantly African American sample of men evaluated in our study differs greatly from the older Finnish women included in the Sipilä and Suominen study [13], other important study differences that may have contributed to differences in findings include the ROI examined within the anterior thigh musculature, echogenicity measurement methods, and the muscle mass HU cutoff values used to denote normal density muscle tissue. The HU cutoff values used in the current study were those established by Goodpaster et al. [35] (+31 to +100 HU), whereas the cutoff values used by Sipilä and Suominen [13] (0 to +150 HU) were determined via their pilot work and informed by studies that preceded their investigation [66].

Recent studies have served to validate the use of muscle echogenicity to assess muscle tissue composition using reference values from MRI [17,67]. Young et al. [67] evaluated echogenicity and MRI estimates of IMAT in four lower extremity muscle groups in a sample of adult men (*n* = 14; age: 27.9 ± 14.9 years) and women (*n* = 17; age: 21.9 ± 2.5 years). The rectus femoris was the common muscle group featured in both this report and the study conducted by Young et al. [66]. The echogenicity of the rectus femoris was significantly associated with IMAT estimates derived from the determined voxel intensity obtained from the same region using T1-weighted MRI images. However, the approach used in the current report was to examine the IMAT for the entire musculature of the mid-thigh using axial CT scans. The proportion of IMAT identified at the mid-thigh CSA was a median value of 17.1% (IQR: 14.18%, 22.56%) for our sample. The proportions of lower extremity IMAT reported in this work and by Young et al. [66] are comparable, with the modestly higher values reported by our group being influenced by the reference imaging modality, older study participants, and use of mid-thigh CSA tissue segmentation inclusive of all muscle groups. Additionally, the strength of association between echogenicity and IMAT via MRI is comparatively higher in the results reported by Young et al. [67] given their use of corresponding ROIs for individual lower extremity muscle groups. In contrast, a key objective of the current work was to understand whether the echogenicity detected within the limited field of view provided by the rectus femoris scan could broadly reflect mid-thigh CSA IMAT based on CT imaging. While there are important methodological differences between our investigation and the aforementioned validation studies using CT and MRI, they all collectively support the use of muscle tissue echogenicity as a viable surrogate measure of muscle quality. 

### 5.3. Quantitative Ultrasound Muscle Measures Are Linked to Tissue Properties and Health Outcomes

Other investigators have recently reported compelling links between muscle tissue properties and echogenicity. Akima et al. [17] recently provided evidence suggesting that lower extremity echogenicity values largely reflect extramyocellular lipids in a sample of young (*n* = 15; age: 20.9 ± 0.3 years) and old adults (*n* = 15; age: 70.7 ± 3.8 years) based on ^1^H MRS analysis. Vastus lateralis and biceps femoris echogenicity exhibited moderate associations with ^1^H MRS estimates of extramyocellular lipids (*r* = 0.49, *p* < 0.01, and *r* = 0.65, *p* < 0.001, respectively), but the associations with intramyocellular lipids were non-significant. Using a histochemical approach to characterize muscle tissue composition, Choi et al. [68] obtained vastus lateralis biopsy samples from older participants, and analyzed muscle fibers for intramyocellular lipids in a subset of 10 participants (normal weight, *n* = 5; obese, *n* = 5), with the obese subgroup displaying 2.4-fold more intramyocellular lipids than in the normal weight subgroup [68]. The standard deviation (SD) of the quadriceps echogenicity values was positively associated with the proportion of intramyocellular lipids (*r* = 0.75, *p* = 0.02) and negatively associated with single-fiber specific force (*r* = −0.74, *p* = 0.02). In reviewing the discordant findings of Akima et al. [17] and Choi et al. [68], it should be noted that extramyocellular lipid spectroscopy signals are position dependent and their discrete distribution in proximity to muscle fasciculi will result in varied signal amplitudes depending on location, whereas intramyocellular lipid spectroscopy signals are relatively impervious to minor voxel translocations [69,70]. Moreover, the significant relationship between echogenicity and intramyocellular lipids reported by Choi et al. [68] may be partially explained by their inclusion of obese participants and their use of grayscale histogram dispersion data to characterize the quadriceps ROI. In considering the findings of this report and the aforementioned studies of Akima and Choi, surrogate measures of muscle quality appear to have utility in characterizing muscle morphology and performance in clinical settings.

The relationship of muscle tissue composition with biomarkers of cardiometabolic health is an active area of study [24,71], but is less well studied using sonographic methods in comparison to CT methods. Findings from the Framingham Heart Study cohort (*n* = 2945) involving older men (age: 49.6 ± 10.7 years) and women (age: 52.0 ± 9.8 years) suggested that CT estimates of IMAT are significantly associated with certain metabolic measures. For example, both HDL (*p* < 0.0001) and triglycerides (*p* < 0.001) were associated with CT attenuation of the paraspinous muscles after adjusting for BMI and visceral fat in men [71]. In the current report, muscle echogenicity and CT estimates of IMAT were both significantly associated with HDL and postprandial glucose values results in our sample of older men. These relationships were similar when controlling for covariates of age and body fat percentage in a multiple linear regression model for predicting postprandial glucose values. However, the strength of the relationship between echogenicity and HDL was diminished when controlling for both covariates. This result may reflect the limited field of view for the rectus femoris echogenicity measures in comparison to the full mid-thigh CSA IMAT estimate derived from CT attenuation values. Nevertheless, the significant relationship between echogenicity and the postprandial glucose values (*r* = 0.43, *p* = 0.018) in our report is strengthened by the prior observation that extramyocellular lipids are negatively associated with insulin sensitivity (*r* = −0.53, *p* < 0.05) even in very young study participants [72]. Estimates of muscle tissue composition using mid-thigh CT attenuation may exhibit an independent association with insulin resistance [73], but this relationship is modified by participant sex and racial/ethnic background [74]. In considering the participant demographics of our sample, it should be noted that higher estimates of IMAT have been reported in lean diabetic versus non diabetic older men of African ancestry (*p* < 0.01) [48,74]. These significant differences in IMAT were retained even after adjusting for age, stature, total muscle area, and total fat [48]. These collective findings suggest that surrogate measures of muscle quality may inform the clinical management of individuals with metabolic disorders and age-related muscle dysfunction.

### 5.4. Limitations and Future Work

This work has several important limitations that affect the interpretation of the findings. The use of quantitative ultrasound to characterize muscle morphology was limited to echogenicity expressed as grayscale values. However, the findings of this report do not suggest that muscle morphology features besides echogenicity are not important contributors to physical performance in older adults [59,75]. Rather, the findings support the inclusion of muscle composition estimates via echogenicity among the clinical markers used to assess and monitor muscle health in aging adults [11,74]. Additionally, the ability of echogenicity to estimate myosteatosis is likely limited to extramyocellular lipids. Intramyocellular lipids have a stronger inverse relationship with insulin sensitivity than extramyocellular lipids, even after controlling for percent total body fat and abdominal subcutaneous fat [72]. Consequently, the findings in this report may underestimate the relationship between muscle composition and metabolic status. Furthermore, the association of metabolic status with IMAT, body composition, and echogenicity may differ from our findings in older adults with metabolic syndrome.

A single scanning site was used to obtain echogenicity measures in this work. While the mid-thigh is a body region frequently included in muscle composition research [27,28,54], the association between echogenicity and metabolic status may vary with different scanning locations. Importantly, the study participants were ambulatory patients enrolled at an urban U.S. VA medical center and may differ from patients in hospital catchment areas that service rural communities and other geographical locations. Additionally, the findings in this report reflect a sample of predominantly African American participants. Nevertheless, similar relationships between estimates of muscle composition with muscle performance and cardiometabolic health have been observed in diverse patient populations [19,21,71,72]. However, given the impact of age, sex, and nutritional status on total body adiposity and muscle echogenicity, larger studies with appropriate group assignment are needed to account for these factors. The interpretation of data concerning the relationship between muscle composition and performance-based measures would also benefit from a larger study sample. While this work included pragmatic laboratory measures of metabolic status, estimates of metabolic function and associated health-risks would have been better characterized by additional tests and measures. These measures include the waist-to-hip ratio, homeostatic model assessment, glucose clamp technique, or alternate forms of dynamic indices involving postprandial insulin measures.

## 6. Conclusions

It is well known that adverse changes in muscle quality—based on estimates of muscle tissue composition—are associated with diminished strength and poor longitudinal health outcomes [11,23,30,54]. Regrettably, the evidence for estimating muscle composition to assess and monitor muscle health has not been successfully integrated into clinical practice. Barriers to integrating contemporary evidence-based approaches to assessing muscle tissue composition are linked to the radiation exposure concerns associated with CT scanning, the limitations of DXA, and cost/access issues regarding MRI [9]. Quantitative ultrasound may provide a safe, low cost, portable, and relatively accessible imaging alternative for the estimation of muscle tissue composition. In this report, construct validity of ultrasound was examined based on the association of rectus femoris echogenicity with mid-thigh CT scan estimates of IMAT, basic metabolic parameters via blood sample analysis, muscle strength, and mobility status. Our findings in a sample of older, predominantly African American men indicate that rectus femoris echogenicity is strongly associated with CT scan estimates of IMAT at the mid-thigh. Moreover, our data suggest that echogenicity and CT scan estimates of IMAT are similarly associated with postprandial glucose values and HDL values, as well as hand grip strength, and knee extension (180°/s) strength values adjusted for body size. Given the similar associations of mid-thigh echogenicity and CT scan estimates of IMAT with physical performance and metabolic parameters in our older male participants, echogenicity should be further considered as a surrogate measure of muscle quality.

## Figures and Tables

**Table 1:** Participant characteristics.

Participant Characteristics (*n* = 30)	
Age (years)	62.5 ± 9.2
Racial/ethnic group	
African American	24 (80.0%)
Caucasian	6 (20.0%)
Body mass index	26.3 ± 3.8
Echogenicity (GSL) ^a^	31.67 ± 9.41
Body composition ^b^	
aLM/ht^2^ (kg/m^2^)	8.57 ± 1.12
Body fat (%)	27.8 ± 7.4
Muscle tissue composition (mid-thigh CSA) ^c^	
Lean mass (%)	53.1 ± 12.1
Subcutaneous fat (%)	28.6 ± 8.3
IMAT (%)	17.1 (14.18, 22.56)
Muscle strength ^d^	
Grip strength 0°/s (kg)	39.5 ± 9.2
Grip strength 0°/s (Adj.)	0.49 ± 0.98
Knee extensors 60°/s (Nm)	194.8 ± 65.7
Knee extensors 60°/s (Adj.)	0.58 ± 0.16
Knee extensors 180°/s (Nm)	126.2 ± 50.7
Knee extensors 180°/s (Adj.)	0.37 ± 0.12
Mobility	
Customary gait speed (m/s)	1.23 ± 0.34
Fast gait speed (m/s)	1.62 ± 0.41
Metabolic parameters	
OGTT (mg/dL) ^e^	105.00 (77.75, 123.50)
Insulin (mg/dL)	3.74 (2.72, 6.78)
Glucose (uM/mL)	92.00 (88.00, 99.25)
QUICKI	0.39 (.36, 0.41)
TC (mg/dL)	185.15 ± 37.79
LDL (mg/dL)	114.50 ± 36.80
HDL (mg/dL)	54.45 ± 13.48
TG (mg/dL)	81.92 ± 32.99

Parametric data are expressed as mean values ± SD, and non-parametric data are expressed as median values and interquartile range. GSL, grayscale levels (unitless); aLM, appendicular lean mass; CSA, cross-sectional area; IMAT, intra- and intermuscular adipose tissue; Adj., adjusted; OGTT, oral glucose tolerance test; QUICKI, quantitative insulin sensitivity check index; TC, total cholesterol; HDL, high-density lipoproteins, LDL, low-density lipoproteins; TG, triglycerides. ^a^ Echogenicity obtained from the rectus femoris of the dominant limb. ^b^ Body composition estimated using dual-energy X-ray absorptiometry (DXA). ^c^ IMAT and subcutaneous fat values derived from computed tomography attenuation at the mid-thigh of the dominant limb. ^d^ All strength values reported for the dominant limb with the adjusted values scaled for body weight. ^e^ OGTT two-hour postprandial glucose values.

**Table 2:** Associations among surrogate measures of muscle quality and body composition estimates.

		Echogenicity ^a^	IMAT ^b^	Subcutaneous Fat ^b^	Total Body Fat ^c^	Lean Body Mass ^d^
Echogenicity ^a^	*r*	1	0.73	0.47	0.51	−0.29
	*p-value*		<0.001 *	0.008 *	0.004 *	0.126
IMAT ^b^	*r*		1	0.38	0.54	0.03
	*p-value*			0.038 *	0.002 *	0.882
Subcutaneous fat ^b^	*r*			1	0.62 *	0.11
	*p-value*				<0.001	0.553
Total body fat ^c^	*r*				1	0.18
	*p-value*					0.328
Lean body mass ^d^	*r*					1
	*p-value*					

IMAT, intra- and intermuscular adipose tissue; Adj., adjusted; Ext., extensors; * *p* < 0.05. ^a^ Echogenicity obtained from the rectus femoris of the dominant limb and measured via grayscale histogram analysis. ^b^ IMAT and subcutaneous fat values derived from computed tomography attenuation at the mid-thigh of the dominant limb; log transformed data used in the analysis. ^c^ Total body fat estimated using dual-energy X-ray absorptiometry (DXA). ^d^ Lean body mass is appendicular lean mass scaled to height (aLM/ht^2^).

**Table 3:** Associations among surrogate measures of muscle quality, body composition estimates, and metabolic parameters.

		Insulin ^c^	Glucose ^c^	QUICKI ^c^	OGTT ^c,f^	TC	LDL	HDL	TG
Echogenicity ^a^	*r*	−0.20	−0.33	0.31	0.43	−0.29	−0.13	−0.52	−0.03
	*p-value*	0.438	0.116	0.161	0.018 *	0.114	0.496	0.003 *	0.875
IMAT ^b,c^	*r*	−0.44	−0.14	0.13	0.38	−0.14	0.06	−0.61	0.22
	*p-value*	0.825	0.514	0.57	0.038 *	0.550	0.745	<0.001 *	0.237
Subcutaneous fat ^b^	*r*	0.15	−0.24	−0.17	0.12	0.05	0.14	−0.27	0.02
	*p-value*	0.438	0.255	0.44	0.519	0.799	0.465	0.155	0.937
Total body fat ^d^	*r*	0.06	−0.21	−0.17	0.40	0.06	0.21	-0.059	0.35
	*p-value*	0.751	0.327	0.44	0.029 *	0.738	0.265	0.001 *	0.061
Lean body mass ^e^	*r*	0.17	0.46	−0.28	−0.13	0.30	0.33	−0.11	0.17
	*p-value*	0.401	0.023 *	0.20	0.503	0.111	0.076	0.574	0.382

QUICKI, Quantitative Insulin Sensitivity Check Index; OGTT, oral glucose tolerance test; TC, total cholesterol; HDL, high-density lipoproteins, LDL, low-density lipoproteins; TG, triglycerides; IMAT, intra- and intermuscular adipose tissue; * *p* < 0.05. ^a^ Echogenicity obtained from the rectus femoris of the dominant limb and measured via grayscale histogram analysis. ^b^ IMAT and subcutaneous fat values derived from computed tomography attenuation at the mid-thigh of the dominant limb. ^c^ Log transformed data used in the analysis. ^d^ Total body fat estimated using dual-energy X-ray absorptiometry (DXA). ^e^ Lean body mass is appendicular lean mass scaled to height (aLM/ht^2^). ^f^ Two-hour postprandial glucose values.

**Table 4:** Associations between measures of muscle quality, physical performance and mobility.

		Grip	Adj. Grip	60°/s Knee Ext.	Adj. 60°/s Knee Ext.	180°/s Knee Ext.	Adj. 180°/s Knee Ext.
Echogenicity ^a^	*r*	−0.41	−0.50	−0.38	−0.47	−0.41	−0.49
	*p-value*	0.026 *	0.005 *	0.038 *	0.008 *	0.025 *	0.006 *
IMAT ^b^	*r*	−0.13	−0.45	−0.14	−0.34	−0.21	−0.40
	*p-value*	0.496	0.013 *	0.476	0.069	0.275	0.029 *
Subcutaneous fat ^b^	*r*	0.12	−0.16	0.14	−0.02	0.06	−0.10
	*p-value*	0.519	0.388	0.451	0.909	0.760	0.612
Total body fat ^c^	*r*	0.05	−0.31	0.20	−0.02	0.12	−0.07
	*p-value*	0.809	0.091	0.285	0.921	0.527	0.709
Lean body mass ^d^	*r*	0.50	0.00	0.53	0.24	0.49	0.21
	*p-value*	0.005 *	0.992	0.002 *	0.198	0.006 *	0.274

^1^ All strength values reported for the dominant limb with the adjusted values scaled for body weight; * *p* < 0.05. IMAT, intra- and intermuscular adipose tissue; Adj., adjusted; Ext., extensors. ^a^ Echogenicity obtained from the rectus femoris of the dominant limb and measured via grayscale histogram analysis. ^b^ IMAT and subcutaneous fat values derived from computed tomography attenuation at the mid-thigh of the dominant limb; log transformed data used in the analysis. ^c^ Total body fat estimated using dual-energy X-ray absorptiometry (DXA). ^d^ Lean body mass is appendicular lean mass scaled to height (aLM/ht^2^).

**Table 5:** Differences in muscle strength based on the first and third tertiles of rectus femoris echogenicity and mid-thigh intra- and intermuscular adipose tissue estimates.

		Peak Grip Force		Peak Knee Extensor Torque			
		0°/s (kg)	Adj. 0°/s	60°/s (Nm)	Adj. 60°/s	180°/s (Nm)	Adj. 180°/s
IMAT: 1st and 3rd tertiles (*df* = 19)							
<15.7%	Mean ± SD	40.3 ± 4.8	0.54 ± 0.07	194.2 ± 53.9	0.61 ± 0.14	129.7 ± 48.4	0.40 ± 0.12
≥20.0%		36.6 ± 9.9	0.44 ± 0.10	166.3 ± 61.4	0.49 ± 0.16	99.2 ± 40.3	0.29 ± 0.11
	MD 95% CI	−3.4–10.6	0.02–0.18	−24.7–80.6	0.01–0.26	−10.5–71.36	0.01–0.22
	SE	3.4	0.04	25.2	0.07	19.6	0.05
	*t*	1.09	2.63	1.11	1.90	1.56	2.21
	*p*-value	0.290	0.016 *	0.281	0.076	0.136	0.040 *
Echo: 1st and 3rd tertiles (*df* = 18)							
<28.77	Mean ± SD	45.4 ± 7.0	0.55 ± 0.08	224.7 ± 56.1	0.64 ± 0.07	153.3 ± 53.0	0.43 ± 0.11
≥34.54		36.4 ± 8.2	0.46 ± 0.09	153.3 ± 59.0	0.47 ± 0.18	98.7 ± 34.0	0.31 ± 0.11
	MD 95% CI	1.9–16.2	0.01–0.17	17.3–125.4	0.04–0.32	7.2–92.0	0.02–0.22
	SE	3.4	0.04	25.7	0.06	20.2	0.05
	*t*	2.64	2.43	2.77	2.94	2.46	2.50
	*p*-value	0.016 *	0.026 *	0.013 *	0.012 *	0.024 *	0.022 *

^1^ All strength values reported for the dominant limb with the adjusted values scaled for body weight; * *p* < 0.05. IMAT, intra- and intermuscular adipose tissue; Echo, echogenicity; Adj., adjusted; Ext., extensors. ^a^ Echogenicity obtained from the rectus femoris of the dominant limb and measured via grayscale histogram analysis. ^b^ IMAT values derived from computed tomography attenuation at the mid-thigh of the dominant limb; log transformed data used in the analysis.
